# Green synthesis and characterization of Bi_2_O_3_ nanoparticles as outstanding filler in polyester for radiation shielding performance

**DOI:** 10.1038/s41598-025-26415-0

**Published:** 2025-11-18

**Authors:** Mohamed A. Hamada, Olfat M. Sadek, W. K. Mekhamer, Ibrahim H. Saleh, M. Elsafi

**Affiliations:** 1 Materials Science Department, Institute of Graduate Studies and Research, Alexandria University, Alexandria, Egypt; 2 Environmental Studies Department, Institute of Graduate Studies and Research, Alexandria University, Alexandria, Egypt; 3 Physics Department, Faculty of Science, Alexandria University, Alexandria, 21511 Egypt

**Keywords:** Green synthesis, Bismuth oxide, Unsaturated polyester, Gamma-rays, Shielding, Thermal properties, Chemistry, Environmental sciences, Materials science, Nanoscience and technology

## Abstract

In this work, Bismuth oxide nanoparticles (Bi_2_O_3_ NPs) were prepared by green synthesis method as cost effective and ecofriendly method using olive leaves extract. The successful preparation of nanoparticles was confirmed using IR and XRD. The Bi_2_O_3_ NPs were used in preparation of unsaturated polyester nanocomposites with different ratios (1, 5, 10, and 20 wt%). The shielding properties of these composites were studied against gamma radiation with different energies emitted from different radioactive sources (59.5, 661.7, (1173 and 1333) keV from Am-241, Cs-137, and Co-60 in series) since different parameters were measured. The results showed that LAC and RSE% increased and HVL and TVL decreased with increase of Bi_2_O_3_ NPs ratio. The 20 wt% nanocomposite sample has highest LAC values among all samples where it has LAC values of 1.668, 0.134, 0.094, and 0.088 cm^− 1^ at 59, 661, 1173, and 1333 keV, respectively. On other hand, pure unsaturated polyester has lowest values where it has LAC values of 0.250, 0.105, 0.078, 0.075 cm^− 1^ at 59, 661, 1173, and 1333 keV, respectively. Also, addition of Bi_2_O_3_ NPs improves thermal properties of the composites where the 20 wt% sample shows 10% weight loss only after 326 °C compared to 247 °C for pure polyester. On other hand, the mechanical properties of unsaturated polyester were negatively affected where compressive strength decreased from 10 to 4.94 MPa with 20 wt% addition percent.

## Introduction

Gamma rays and X-rays are utilized in several applications such as scientific research, industrial applications, medicine, and nuclear power plants^1–4^. However, undesired exposure to ionizing radiation has harmful effects on humans and the environment. It may result in cell alterations, damage to organs, and other health issues^5,6^.

Reduction of absorbed dose can be achieved by control in duration of exposure and remoteness from the radiation source and presence of shields^3,6^. Reinforcement of polymers with high atomic number micro and nano metal oxides enhances its radiation attenuation properties and makes it suitable for use as shields^2,5,7,8^. In addition, polymer composites are flexible and have light weight and good mechanical strength compared to other materials used in shielding^7,9,10^.

One of the metal oxides that is used is bismuth oxide (Bi_2_O_3_) in nano size. Bismuth oxide has a high density and a large atomic number for bismuth (Z = 83). Also, it is non-toxic compared to lead^11^. Many researchers used bismuth oxide nanoparticles in the fabrication of polymer composites for shielding applications. El Sharkawy et al. studied the shielding properties of recycled PVC doped with different percentages of Bi_2_O_3_ NPs at different energies emitted from the Eu-152 source^11^. Also, Yassene et al. prepared HDPE composite reinforced with Bi_2_O_3_ NPs and studied its shielding properties^12^. Elsafi et al. used Bi_2_O_3_ NPs and waste marble with unsaturated polyester and studied the attenuation characteristics for these composites at various energies^13^. Also, Elsafi et al. prepared epoxy composites using Bi_2_O_3_ NPs for shielding applications^14^. Cao et al. improved the shielding properties of PMMA by adding bismuth oxide, and other studies used bismuth oxide with different polymers^5,7,15^.

Unsaturated polyester is a type of thermoset polymer that is used in many applications such as automotive, military products, aircraft, construction, and the marine industry. It is characterized by its low cost, ease of processing, and good mechanical properties^16–19^. Many researchers used unsaturated polyester as a matrix in the composites for radiation shielding applications. Ozdogan et al. added different ratios of PbO to unsaturated polyester and measured its shielding properties^10^. More et al. studied attenuation properties against gamma rays and neutrons for TiO_2_ NPs filled unsaturated polyester composite^20^. Hemily et al. prepared unsaturated polyester composites reinforced with waste marble and WO_3_ NPs for gamma shielding applications^21^. Also, more et al. added SnO_2_ NPs to unsaturated polyester and studied its shielding properties^9^. As well, different studies focused on unsaturated polyester composites for shielding applications^1,22–24^.

Bi_2_O_3_ NPs can be synthesized by various methods such as sol-gel method, co-precipitation, and hydrothermal method, but these methods use toxic chemicals, so it is hazardous to humans and environment. On the other hand, green synthesis is cost-effective, non-toxic, and eco-friendly, as it uses microorganisms and plant parts instead of toxic chemicals. The different plant parts contain a lot of phytochemicals such as flavonoids, phenolic compounds, alkaloids, proteins, and terpenoids which act as reducing agents and stabilizing agents in nanoparticles synthesis^25–27^. Many studies reported the preparation of Bi_2_O_3_ NPs using different plant parts such as leaves of (A) indica plant^28^, (B) multifida plant^29^, leaves of Datura inoxia plant^30^, roots of ginger plant^31^, leaves of Mexican mint plant^32^, S. persica plant^33^, and leaves of Ficus benghalensis plant^26^.

In the present work, Bi_2_O_3_ NPs were prepared by the green synthesis method using olive leaves extract. Then, it used in the fabrication of unsaturated polyester nanocomposites with different filler percentages. The attenuation properties of these composites were studied at different energies in addition to its thermal and mechanical properties.

## Materials and methods

### Materials

Olive leaves were purchased from local market. Bismuth nitrate pentahydrate Bi(NO_3_)_3_ .5H_2_O were obtained from universal fine chemicals Ltd. Glacial acetic acid CH_3_COOH was supplied from fisher scientific. Pre-accelerated unsaturated polyester resin was supplied from SUPIC Co. Methyl ethyl ketone peroxide (MEKP) was supplied from AKPA chemicals.

### Preparation of Olive leaves extract

First, olive leaves were cut into small pieces. Then, 40 gm of chopped leaves were boiled in 500 ml of distilled water at 95 °C for 60 min. After that, the extract was cooled in air, filtered and stored in the refrigerator for further utilization.

### Green synthesis of Bi_2_O_3_ NPs

Initially, 50 ml of 0.1 M Bi(NO_3_)_3_ solution was prepared by dissolution of 2.425 gm Bi(NO_3_)_3_.5H_2_O in 5 ml of acetic acid at 60 °C. Then, it was completed to 50 ml with distilled water. This solution was added dropwise to 400 ml of olive leaves extract at 60 °C under vigorous stirring. The mixture was stirred for 3 h under the same conditions. After this period, the resultant precipitate was filtered, washed several times with distilled water, then dried in an oven at 70 °C for 4 h and subsequently calcinated in a muffle furnace at 600 °C for 2 h in ambient air.

### Preparation of unsaturated polyester nanocomposites

**Fig. 1:**
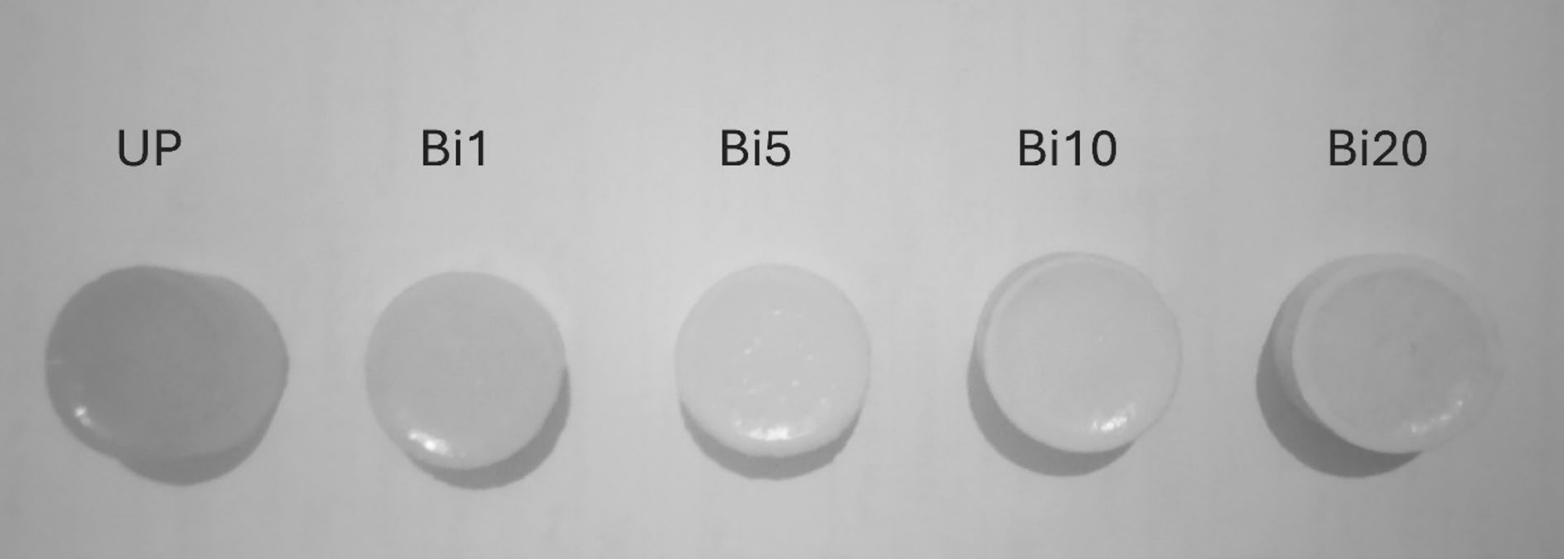
Image of UP and different composites samples.

The Bi_2_O_3_ NPs with different wt % (0, 1, 5, 10, and 20) were mixed with unsaturated polyester resin followed by sonication for 30 min. Thereafter, the mixture was stirred for 30 min. Then, 1% of MEKP was added to the mixture to initiate curing reaction. Finally, the mixture was poured into cylindrical molds and allowed to solidify. Table 1; Fig. 1 show the composite samples and its composition and density. The mass per volume law was used to practically estimate the samples’ density. A digital balance with a ± 1 mg precision was used to measure the mass, and the (x πr^2^) formula was used to calculate the volume, where x and r stand for the sample’s thickness and radius, respectively.

**Table 1:** Composition and density of composite samples.

	Composition (%)		Density (gm/cm^3^)
	Unsaturated polyester	Bismuth oxide NPs	
UP	100	0	1.306
Bi1	99	1	1.313
Bi5	95	5	1.351
Bi10	90	10	1.421
Bi20	80	20	1.582

### Characterization

The size and shape of the Bi_2_O_3_ NPs was measured by transmission electron microscope (JEOL JEM-1400 plus). IR spectra of the nanoparticles and nanocomposites were measured by a PerkinElmer spectrum two FT-IR spectrometer in the wave number range of 4000–450 cm^− 1^. XRD patterns were measured by a Bruker XRD D2 phaser diffractometer using Cu Kα radiation (λ = 1.54 Å). Thermal stability of nanocomposites was evaluated with heating rate 10 C^°^/min using Q600 thermogravimetric analyzer, TA instruments. Scanning electron microscope (JEOL JSM IT200) was utilized to study fracture surface of nanocomposites and to make EDX analysis for Bi_2_O_3_ NPs. In addition to, compression test was conducted for nanocomposite samples using universal testing machine (5ST, Tinius Olsen).

### Measurement of attenuation properties

**Fig. 2:**
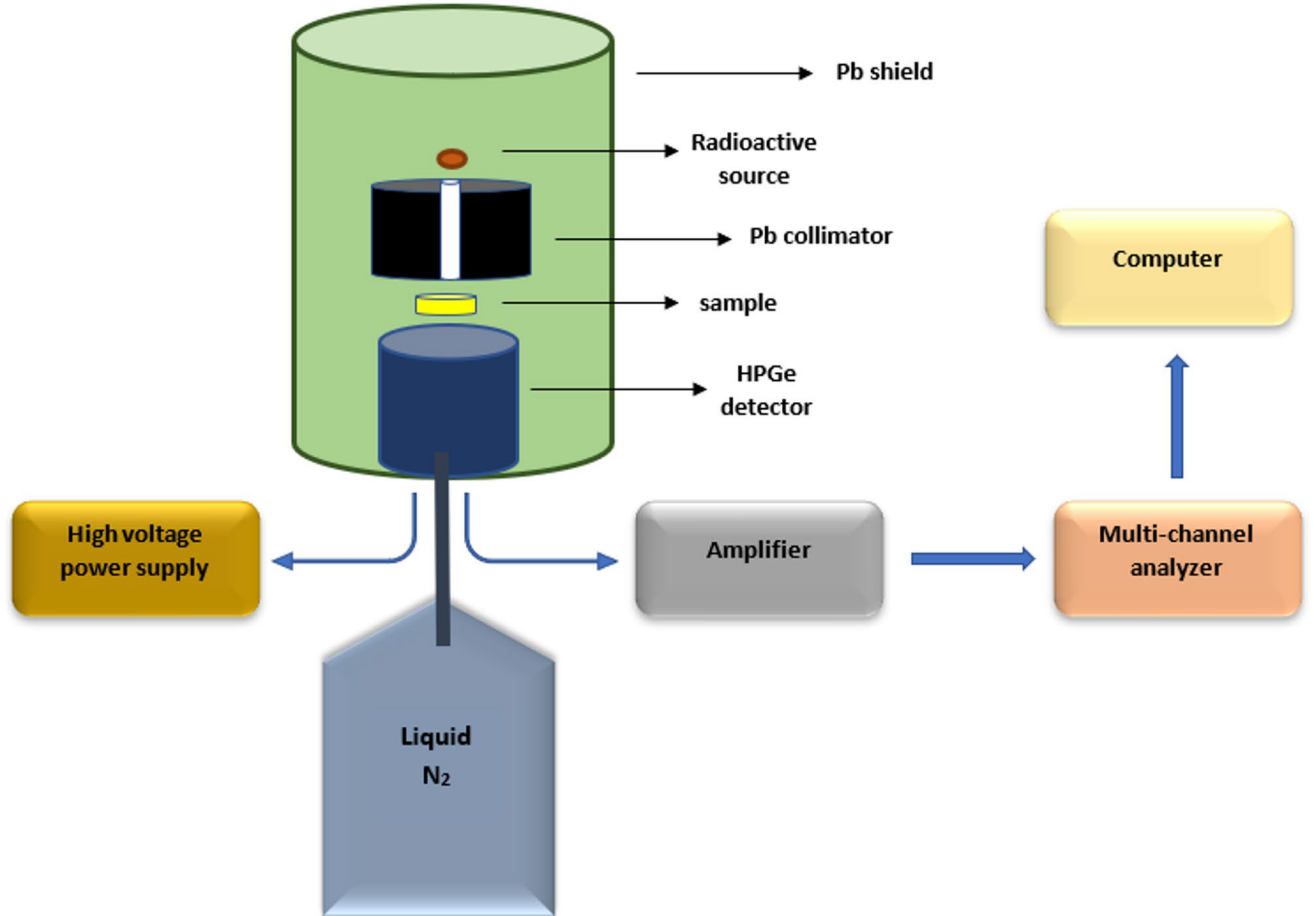
The experimental setup for measurement of attenuation coefficient.

The attenuation characteristics of the composites were examined using a high purity germanium (HPGe) detector with a relative efficiency of 24% and an energy resolution of 1.96 keV at 1333 keV and several radioactive point sources emit gamma rays with different energies (Am-241 emits at 59 keV, Cs-137 emits at 661 keV, and Co-60 emits at 1173 and 1333 keV) with an initial activity of 40.3 kBq in 1998. The composites samples were placed between the detector and the radioactive source as shown in Fig. 2. The peak intensity was measured in absence and presence of the samples and the linear attenuation coefficient (LAC) was determined at each energy according to Eq. (1)

1 $${\text{LAC}}={\text{ln}}\left( {{{\text{I}}_{\text{o}}}/{\text{I}}} \right)/{\text{x}}$$

Where, I_0_, I, and x represent peak intensity in absence of sample, peak intensity in presence of sample, and thickness of sample, respectively. From LAC values, other parameters of shielding such as half value layer (HVL), tenth value layer (TVL), and radiation shielding efficiency (RSE%) were be measured using Eqs. (2), (3), and (4)^6,34^.

2 $${\text{HVL}}={\text{ln(2)}}/{\text{LAC}}$$

3 $${\text{TVL}}={\text{ln(1}}0{\text{)}}/{\text{LAC}}$$

4 $${\text{RSE}}\% =\left( {{\text{1}}-{\text{I}}/{{\text{I}}_{\text{o}}}} \right)*{\text{1}}00$$

## Results and discussion

### TEM and EDX analysis of Bi_2_O_3_ NPs

**Fig. 3:**
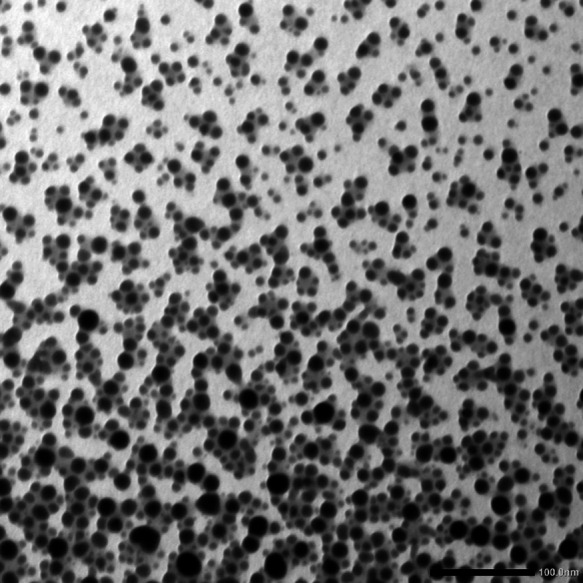
TEM image of pure Bi_2_O_3_ NPs synthesized using olive leaves extract.

**Fig. 4:**
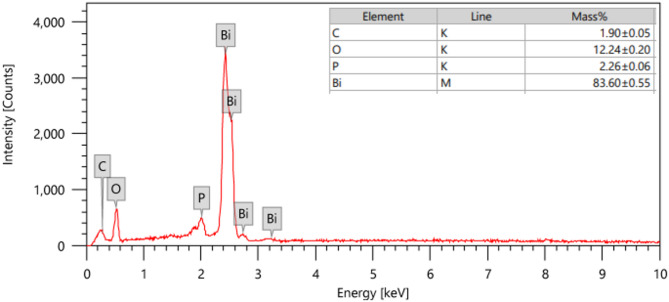
EDX spectrum of Bi_2_O_3_ NPs.

According to illustrated TEM image of Bi_2_O_3_ NPs (Fig. 3), it can be seen that Bi_2_O_3_ NPs have spherical shape with an average particle size of 15 nm. On other hand, EDX analysis (Fig. 4) shows presence of bismuth and oxygen as major elements which confirms the formation of Bi_2_O_3_ with other characterization results in addition to presence of minor amounts of carbon and phosphor which may be residue from calcination step and impurities in starting precursor, respectively.

### FT-IR spectroscopy

**Fig. 5:**
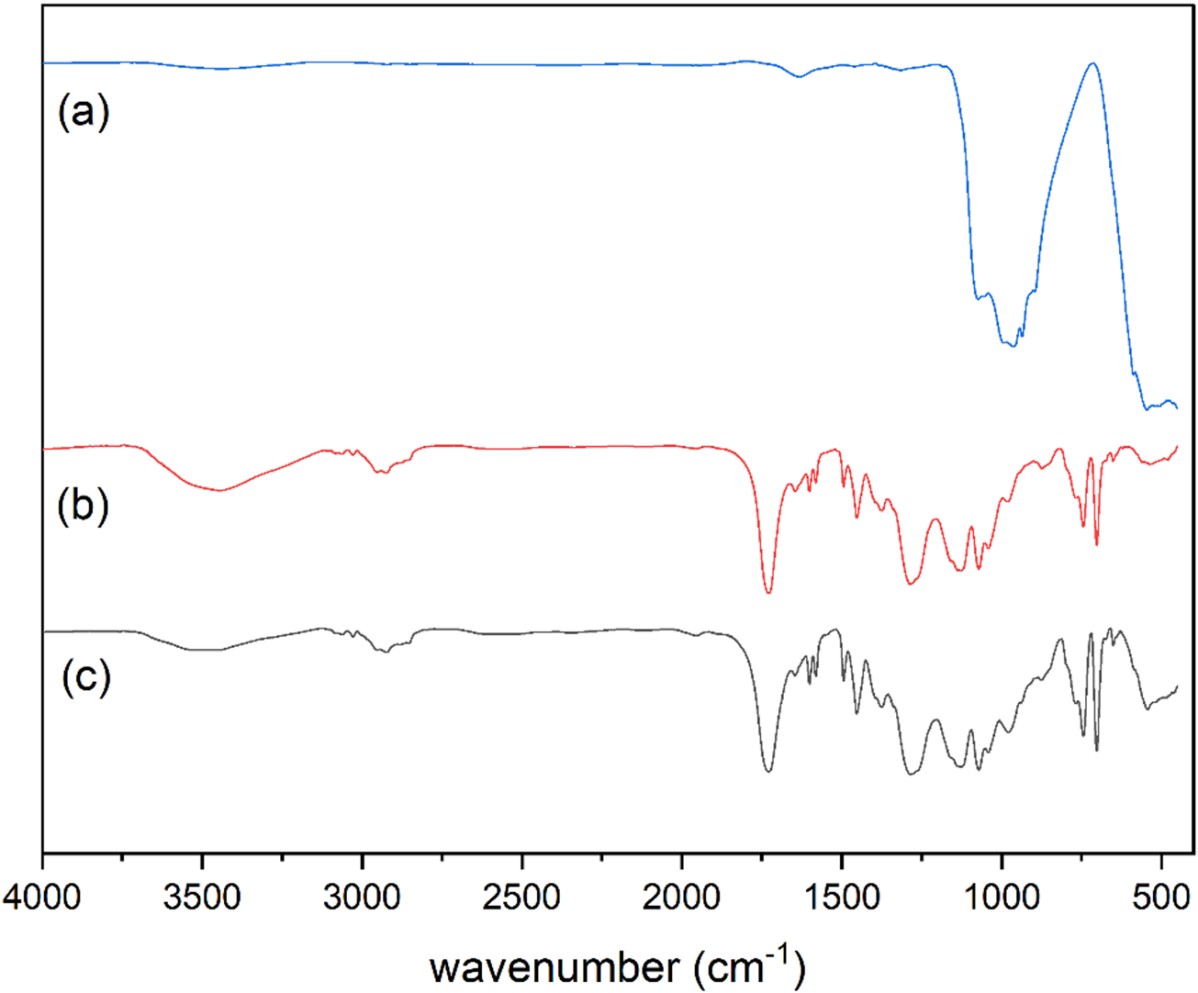
FT-IR spectra of (**a**) Bi_2_O_3_ NPs, (**b**) pure UP, and (**c**) Bi20 nanocomposite.

Figure 5 shows FT-IR spectra of Bi_2_O_3_ NPs, pure UP, and 20 wt% Bi_2_O_3_ nanocomposite. As seen in Fig. 5a, the peak at 546 cm^− 1^ is assigned to metal-oxygen bond (Bi-O-Bi) vibration in Bi_2_O_3_^25,27^. The peaks 937 and 963 cm^− 1^ may be related to Bi-O bond stretching vibrations^25,27,35^. Also, the peak at 1074 cm^− 1^ may be assigned to vibration of Bi-O bond^28,35^. Figure 5b represents the spectrum of pure UP. In which the peak at 3463 cm^− 1^ corresponding to O-H stretching vibration, the peak at 3028 cm^− 1^ is attributed to aromatic C-H bond vibration, and the peak at 2954 cm^− 1^ is related to aliphatic C-H bond stretching. While, the peaks at 1728 and 1453 cm^− 1^ are related to C = O bond stretching vibration and C-H bond bending vibration, respectively. The peaks at 1283, 1130, and 1072 cm^− 1^ are related to C-O bond vibrations and the observed peaks at 745 and 702 cm^− 1^ are related to aromatic C-H bending vibrations^36,37^. In comparing to the spectrum of pure UP (Fig. 5b), it is noticed the presence of new peaks in the spectrum of Bi_2_O_3_/UP composite (Fig. 5c) at 545 and 937 cm^− 1^ related to Bi-O bond which indicates successful addition of Bi_2_O_3_ NPs to unsaturated polyester. Also, it is obvious that there is no shift on the peaks of unsaturated polyester in the composite spectrum which indicates that there is no chemical interaction between the nanoparticles and the matrix^37–39^.

### X-ray diffraction (XRD)

**Fig. 6:**
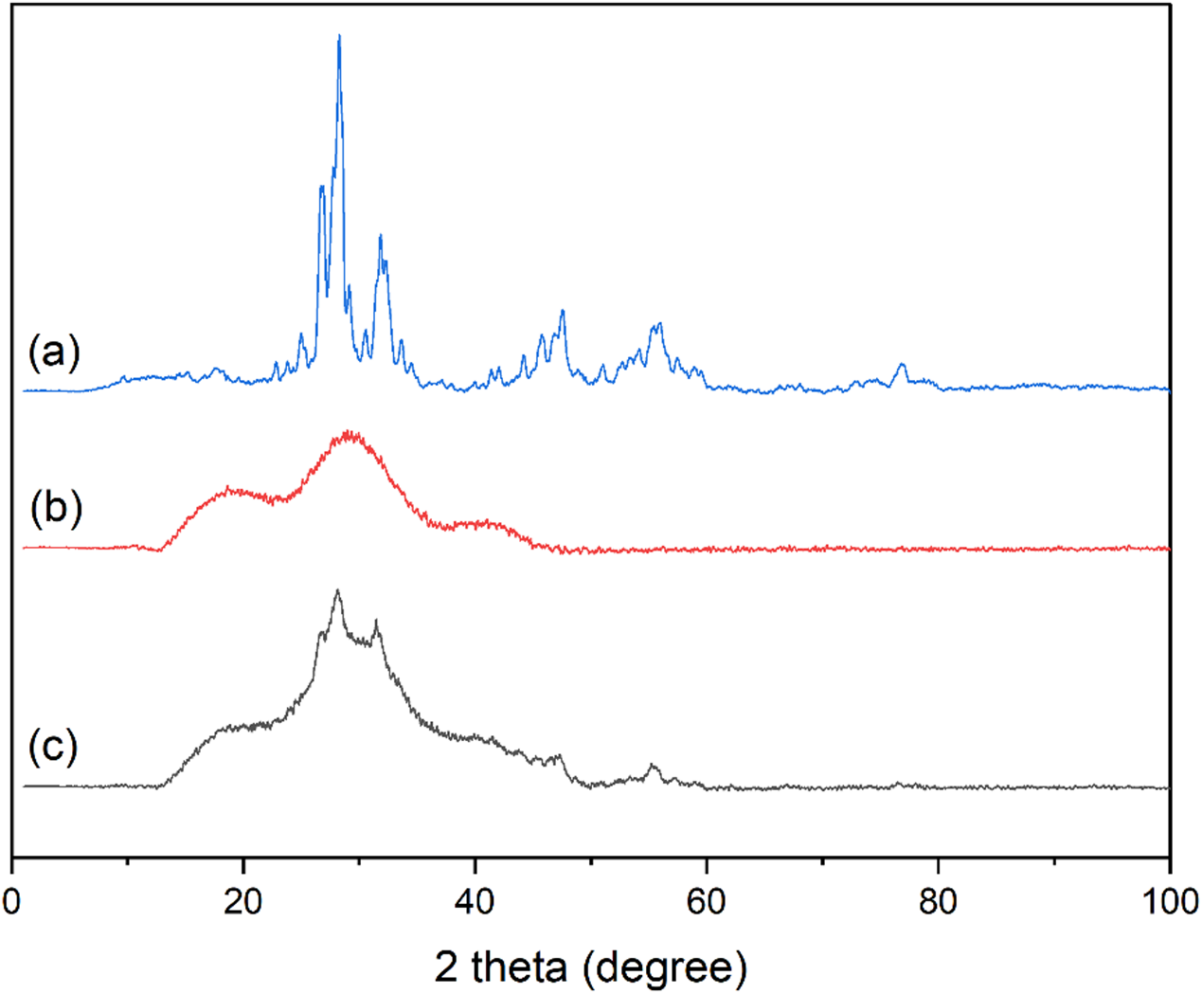
XRD patterns of (**a**) Bi_2_O_3_ NPs, (**b**) pure UP, and (**c**) Bi20 nanocomposite.

Figure 6 Show the XRD patterns of Bi_2_O_3_ NPs, pure UP, and 20% wt Bi_2_O_3_ nanocomposite. As seen in Fig. 6a, there are several peaks in the XRD pattern of Bi_2_O_3_ NPs. The peaks at 2θ values of 24.99^°^, 26.89^°^, 27.76^°^, 33.64^°^, 41.40^°^, 42.04^°^, 44.16^°^, 46.83^°^, 55.42^°^, and 58.91^°^ which corresponding to (102), (111), (120), (200), (131), (122), (040), (041), (241), and (024) diffraction planes which can be attributed to monoclinic Bi_2_O_3_ (JCPDS card 41-1449) with the following lattice parameters: a = 5.849 Å, b = 8.169 Å, c = 7.512 Å, α = γ = 90^°^, and β = 112.96^°^^25,30,31,33,35,40,41^. While the peaks at 2θ values of 28.28^°^, 30.53^°^, 31.84^°^, 32.30^°^, 45.75^°^, 47.54^°^, 54.13^°^, 55.96^°^, and 57.46^°^ corresponding to (201), (211), (002), (220), (222), (400), (203), (421), and (402) diffraction planes of tetragonal Bi_2_O_3_ (JCPDS cards 27–0050) with the following lattice parameters: a = b = 7.73 Å, c = 5.64 Å, and α = β = γ = 90^°^^25,35,42,43^. On other hand, the peak at 2θ value of 29.16^°^ may be attributed to non-stoichiometric phase of Bi_2_O_3_^44^. Also, the peak at 2θ value of 51.01^°^ can be assigned to (023) diffraction plane in BiPO_4_ which confirm with presence of phosphor element in EDX analysis^45^. The average crystallite size of Bi_2_O_3_ NPs is calculated and it is found to be 12.98 nm using Scherrer’s Eqs.^25,29^ :

$$\:D=\:\frac{K\lambda\:}{\beta\:cos\theta\:}$$

Where *D* is the crystallite size, K is the shape factor (taken as 0.9), λ is the X-ray wavelength (1.54 Å for Cu Kα radiation), β is the full width at half maximum (FWHM) of the diffraction peak, and θ is the Bragg’s diffraction angle. Table 2 shows FWHM values and other XRD parameters for each peak.

**Table 2:** XRD parameters of synthesized Bi_2_O_3_ nanoparticles.

2θ	Hkl	FWHM	Size (nm)
26.89	111	0.5367	15.21
28.28	201	2.0523	3.99
30.53	211	0.5631	14.62
31.84	002	0.9802	8.42
32.30	220	0.5539	14.93
33.64	200	1.71	4.84
46.83	041	0.4542	19.06
47.54	222	0.5061	17.15
55.42	241	0.4728	18.96
55.96	421	0.6140	14.64

It is noticed in Fig. 6b. that there are broad peaks at 2θ values of 19.33^°^, 28.74^°^, and 40.64^°^ which indicates the amorphous nature of unsaturated polyester. On other hand, XRD pattern of the nanocomposite (Fig. 6c.) show the decrease in intensity of broad peaks and appearance of sharp peaks at 2θ values of 26.73^°^, 28.14^°^, 31.49^°^, 47.22^°^, and 55.30^°^ related to Bi_2_O_3_ NPs which reflects the slight increase in crystallinity with addition of Bi_2_O_3_ NPs^20,46–48^.

### Thermal gravimetric analysis (TGA)

**Fig. 7:**
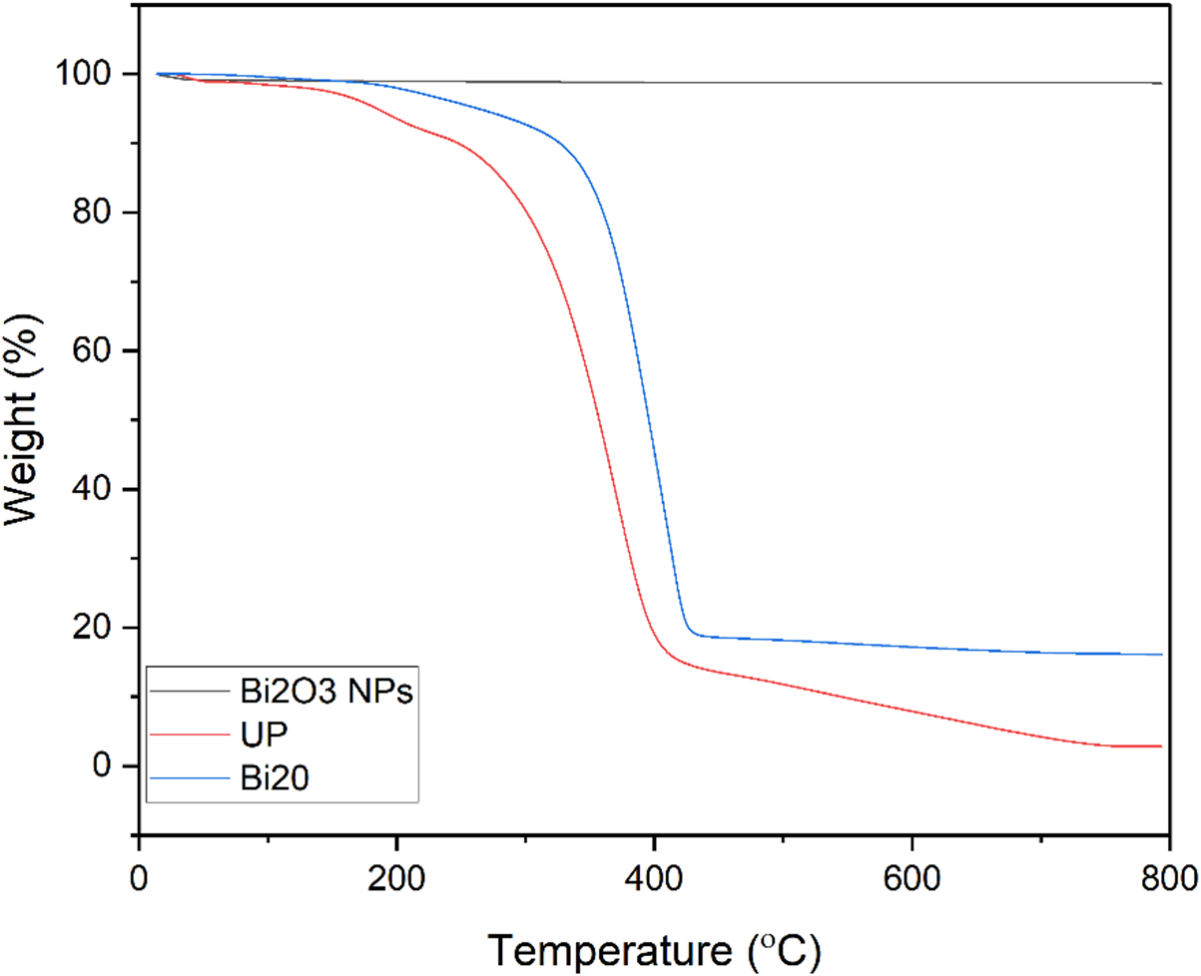
TGA curve of Bi_2_O_3_ NPs, pure UP, and Bi20 nanocomposite.

Figure 7 Shows the TGA curve of Bi_2_O_3_ NPs, pure UP, and 20% wt Bi_2_O_3_ nanocomposite. It is noticed that Bi_2_O_3_ NPs have high thermal stability up to 800 °C where it lost about 1.33% from its weight only. This very small weight loss may be because of the evaporation of adsorbed water on the surface of Bi_2_O_3_ NPs^11^. Also, it is seen that pure UP lost 10% of its weight at 247 °C and lost 50% of its weight at 357 °C. On other hand, the 20% wt nanocomposite degraded at higher temperatures where it lost 10% of its weight at 326 °C and lost 50% of its weight at 395 °C which indicates that the addition of Bi_2_O_3_ NPs retards the thermal decomposition of unsaturated polyester and improves its thermal stability^49^.

### Scanning electron microscope (SEM)

**Fig. 8:**
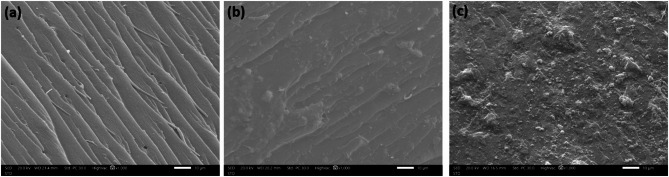
SEM images of (**a**) pure UP, (**b**) Bi5 nanocomposite, and (**c**) Bi20 nanocomposite.

Figure 8 Shows SEM images of fracture surfaces of pure UP, 5% wt nanocomposite, and 20% wt nanocomposite. Figure 8a. shows a smooth fracture surface for pure UP which indicates the brittle fracture of unsaturated polyester. While, Fig. 8b and c show rough fracture surfaces for Bi5 and Bi20 nanocomposites which indicate that the nanocomposites have some toughness. Also, at higher loading, it is clear there is good dispersion of Bi_2_O_3_ nanoparticles in polymer matrix. However, it is noticed the presence of some agglomerations of nanoparticles in both samples and it increases with the increase of Bi_2_O_3_ NPs ratio^36,49^. Despite the good dispersion of nanoparticles, these agglomerations reduce distribution homogeneity of Bi_2_O_3_ NPs in UP matrix which may affect mechanical properties of the composites as discussed below.

### Compressive strength test

**Fig. 9:**
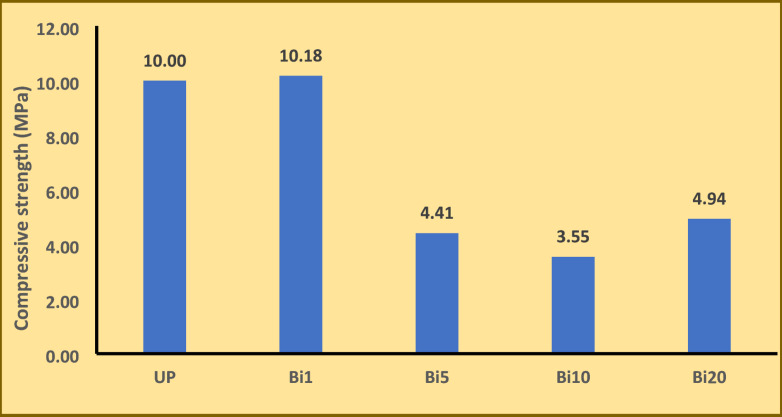
Compressive strength for UP and different nanocomposites.

The mechanical properties of nanocomposites were evaluated through compressive strength test. Figure 9 Shows values of compressive strength for UP and nanocomposites with different percentages of Bi_2_O_3_ NPs. It is noticed the slight increase in compressive strength value for 1% wt nanocomposite which may be due to good dispersion of nanoparticles in composite. Then, the compressive strength decreases with increasing of Bi_2_O_3_ NPs ratio which may be related to agglomeration of nanoparticles and negative effect of nanoparticles on crosslinking reaction during curing process of composite^49,50^.

### Radiation attenuation properties

**Fig. 10:**
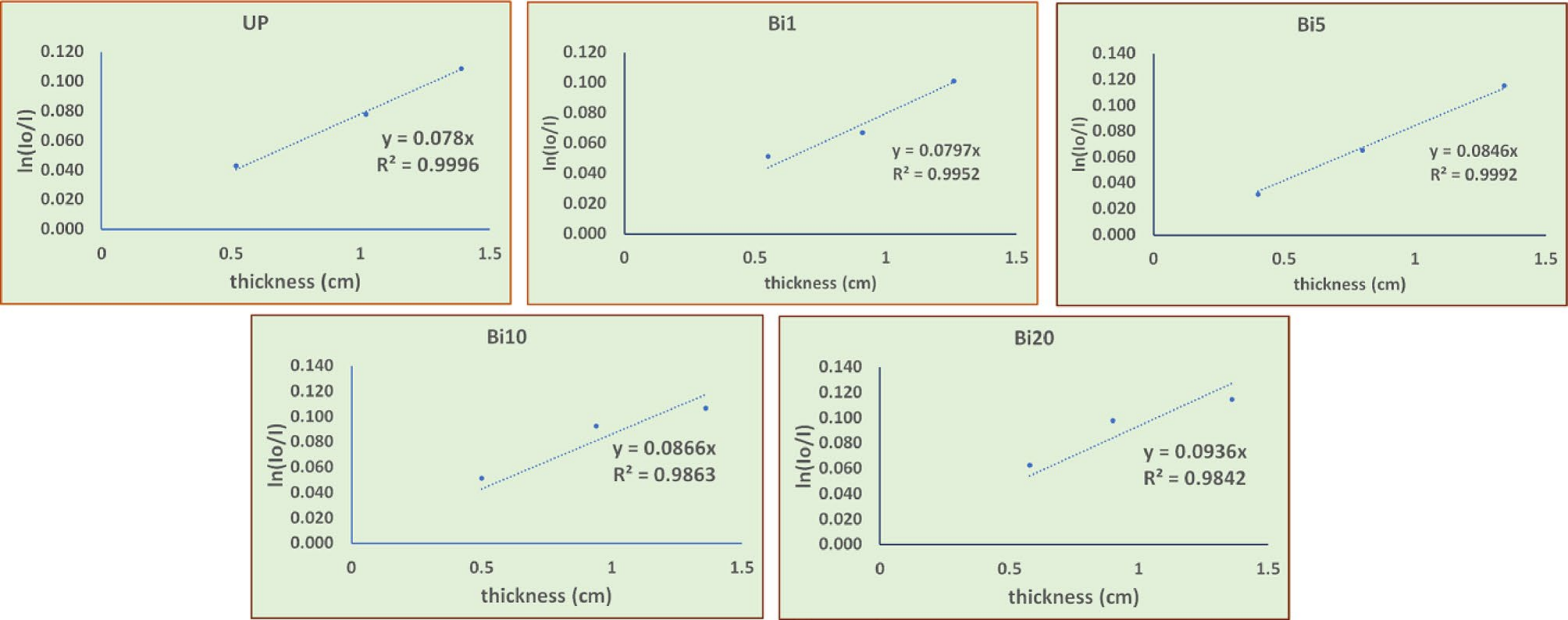
Curve of I_o_ / I against thickness for UP and different composites at 1173 keV.

I_o_ / I ratio was plotted against the thickness (x) for each sample as shown in Fig. 10 and the slope of the curve represents the value of LAC. Table 3 shows the relative deviation between experimental and theoretical values of LAC where the relative deviation was calculated according to the following equation:

$$\:RD\%=\frac{LAC\left(XCOM\right)-LAC(exp.)}{LAC(exp.)}*100$$

It is noticed that relative deviation for all samples doesn’t exceed 6% which indicates compatibility between experimental and theoretical results^51^. The theoretical results were calculated using XCOM software where it is an online software and is used in calculation of attenuation coefficient by entering chemical composition of samples.

**Table 3:** Values of experimental, theoretical LAC and RD% between them.

	59 keV			661 keV			1173 keV			1333 keV		
	Exp.	XCOM	RD%	Exp.	XCOM	RD%	Exp.	XCOM	RD%	Exp.	XCOM	RD%
UP	0.250	0.247	− 0.87	0.105	0.107	2.02	0.078	0.082	4.60	0.075	0.076	2.02
Bi1	0.315	0.310	− 1.74	0.109	0.108	− 0.87	0.080	0.082	2.91	0.077	0.077	0.13
Bi5	0.542	0.568	4.87	0.114	0.113	− 1.24	0.085	0.084	− 0.29	0.078	0.079	0.78
Bi10	0.891	0.927	3.97	0.116	0.120	3.76	0.087	0.089	2.46	0.081	0.083	2.56
Bi20	1.668	1.764	5.72	0.134	0.138	3.18	0.094	0.099	5.49	0.088	0.092	4.68

**Fig. 11:**
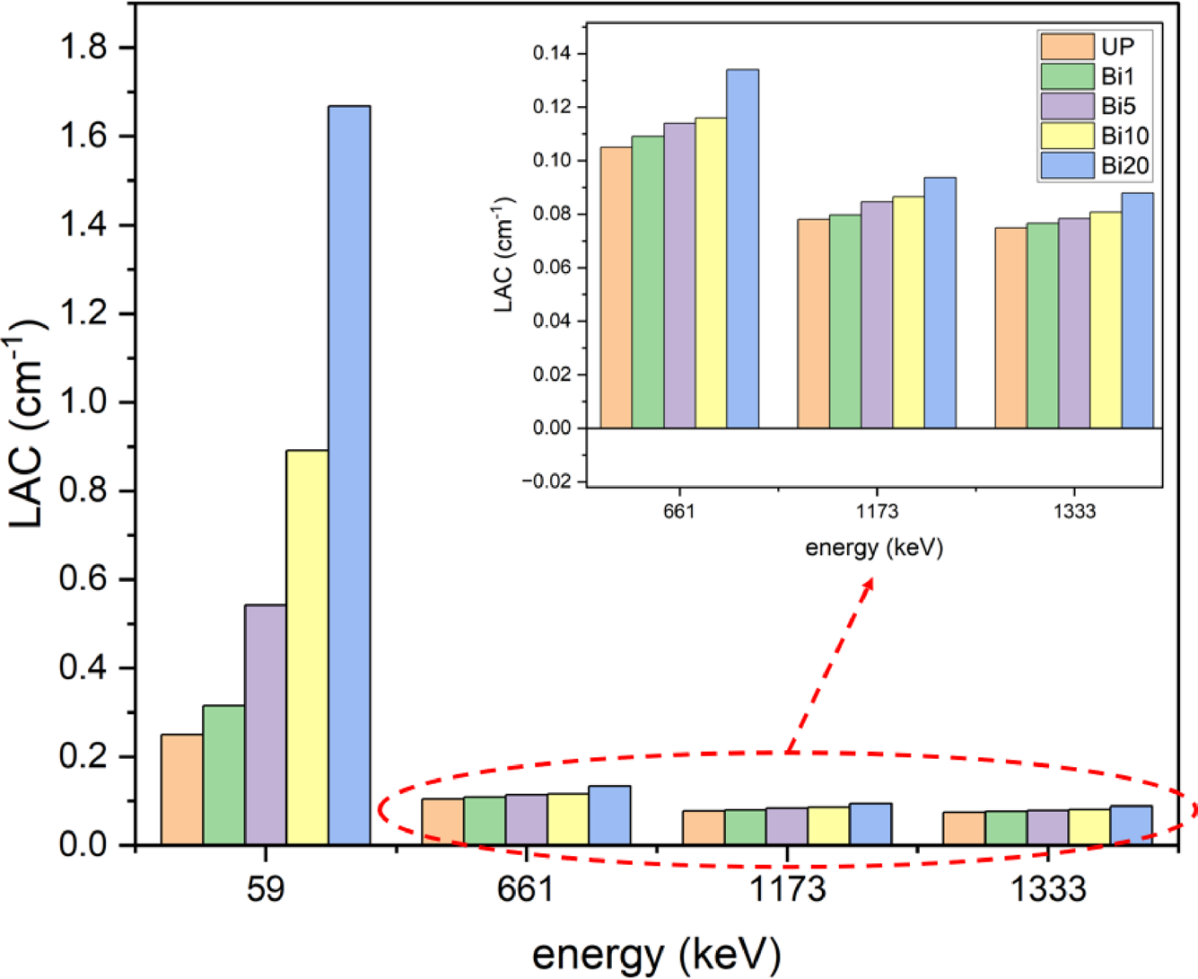
Experimental LAC for UP and different composites at different energies.

Figure 11 shows values of LAC for unsaturated polyester the different composites at different energies. It is observed that LAC has higher values at lower energy (59 keV) and decreases with increase of energy where photoelectric effect is more prevalent at lower energy while Compton scattering and pair production are prevalent at higher energies^7,51,52^. Also, it is noticed the increase of LAC with increase of Bi_2_O_3_ NPs ratio which largely increases from 0.250 cm^− 1^ for pure unsaturated polyester to 1.668 cm^− 1^ for 20 wt% nanocomposite at 59 keV and slightly increases at higher energies due to dominance of photoelectric effect at lower energy.

**Fig. 12:**
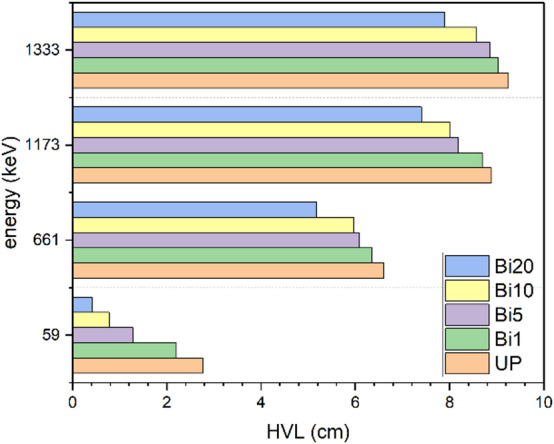
HVL values of UP and different composites at different energies.

**Fig. 13:**
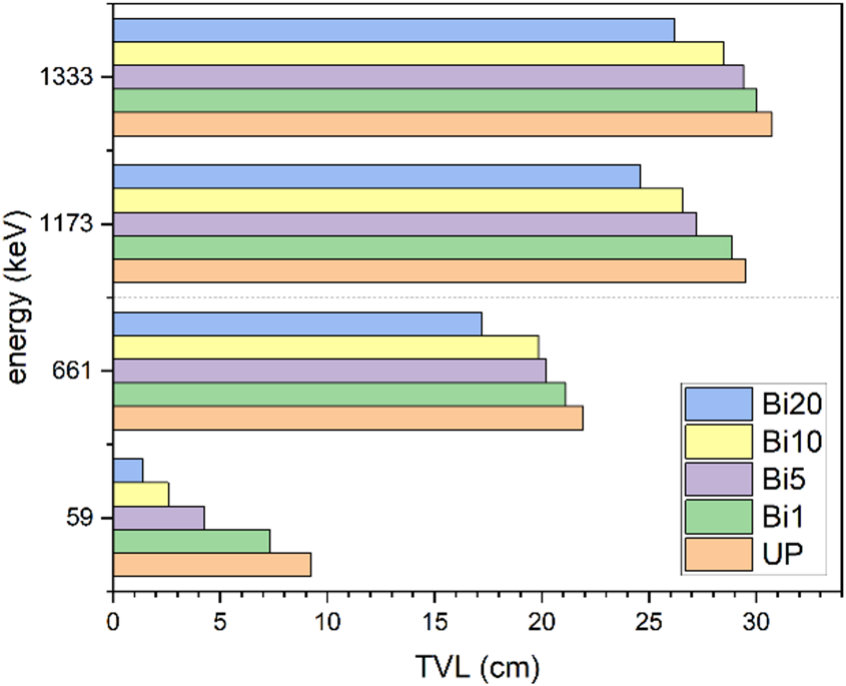
TVL values of UP and different composites at different energies.

**Fig. 14:**
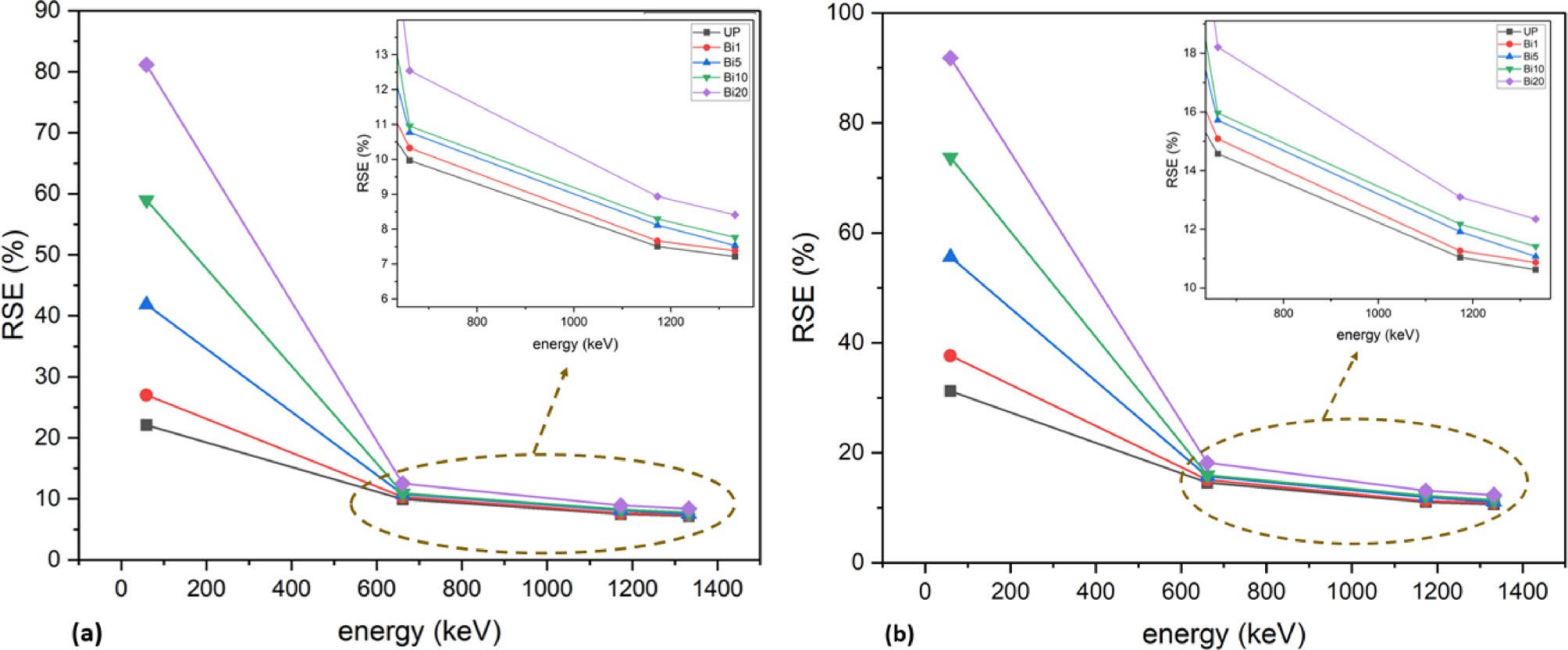
RSE% of UP and different composites at (**a**) 1 cm and (**b**) 1.5 cm thickness.

As it is expected, Fig. 12 shows increase of HVL values with increase of energy where HVL is inversely proportional to LAC and the more thickness is required to prevent higher energy photons from penetrating. It also shows the improvement of HVL values with addition of Bi_2_O_3_ NPs where HVL value of unsaturated polyester improves from 2.77 cm to 0.41 cm at 59 keV with addition of 20% Bi_2_O_3_ NPs. Therefore, a thin layer is enough to absorb half of photons since the photoelectric effect is the dominant at low energy. On other hand, the same addition slightly decreases the HVL value from 9.25 cm to 7.88 cm at 1333 keV where the photoelectric effect becomes less dominant and Compton scattering and pairs production are more dominant at higher energies. As shown in Fig. 13, TVL values have the same trends as HVL values. Also, it is observed that TVL values are greater than HVL values since the more thickness is needed to reduce radiation intensity to tenth of its value. The addition of 20 wt% of Bi_2_O_3_ NPs to unsaturated polyester enhances its TVL value from 9.22 cm to 1.38 cm at 59 keV while it is improved from 30.74 cm to 26.21 cm at 1333 keV which indicates that low thickness of 20 wt% nanocomposites is useful in attenuation of low energy photons. This kind of performance implies that these nanocomposites could be especially helpful for real-world shielding applications. These include lightweight protection panels used in mammography centers, dental radiography offices, and diagnostic X-ray rooms. These composites can also be used in laboratory-scale shielding, where low-energy isotopes are commonly used, and in the creation of building materials for radiation-safe walls and partitions. Their suitability for architectural and biomedical applications is strengthened by the fact that they are lead-free. Additionally, using a green synthesis method lowers its manufacturing cost. Another parameter was be measured is radiation shielding efficiency. Figure 14 shows shielding efficiency at different energies for different thickness. It shows decrease of shielding efficiency with increase of photon energy which is attributed to dominance of photoelectric effect at lower energies as explained in previously discussed parameters. Also, it is observed that 20 wt% nanocomposite has the highest shielding efficiency at 59 keV (81.14% and 91.81% at 1 and 1.5 cm thickness respectively) which reveal its good ability in attenuation of low energy photons.

**Fig. 15:**
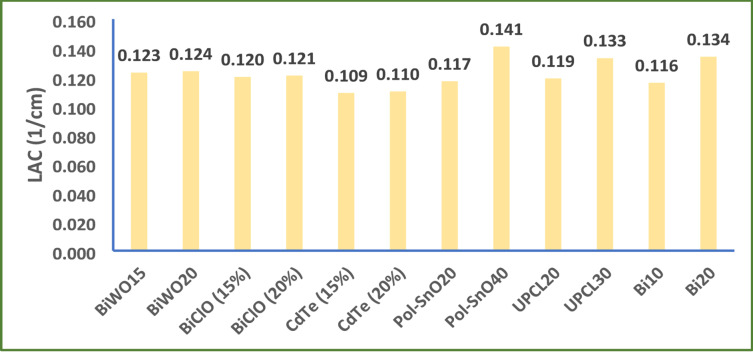
LAC values of different unsaturated polyester composites.

Finally, our results were compared with different previously reported unsaturated polyester composites. Figure 15 Shows LAC values of different unsaturated polyester composites containing Bi_2_(WO_4_)_3_^1^, BiClO^53^, CdTe^2^, SnO^54^, and PbO^49^ and our composites. It shows the agreement of our results with other published results. In addition, it is observed that LAC value of 20 wt% Bi_2_O_3_ nanocomposite is greater than LAC value of the composites containing other bismuth compounds. Also, when 20 wt% Bi_2_O_3_ nanocomposite is compared with PbO composites, it shows greater LAC value which indicates importance of Bi_2_O_3_ as the safe relative for lead compounds in shielding composites.

## Conclusion

In this study, shielding characteristics of unsaturated polyester nanocomposites filled with different ratios of Bi_2_O_3_ NPs were studied. Bi_2_O_3_ NPs were synthesized using olive leaves extract with average particle size of 15 nm as confirmed by TEM image. Also, the results of IR and XRD revealed the good preparation of Bi_2_O_3_ NPs. The LAC, HVL, TVL, and RSE% of the nanocomposites were measured at different energies (59, 661, 1173, and 1333 keV). In general, attenuation properties decreased with increase of energy for all samples. For example, RSE% of unsaturated polyester decreased from 22.09% to 7.22% with increasing of energy from 59 to 1333 keV at 1 cm thickness. On other hand, attenuation properties were improved with increase of Bi_2_O_3_ NPs ratio. The 20 wt% nanocomposite had the best results especially at 59 keV where it has highest LAC value of 1.668 cm^− 1^ and lowest HVL value of 0.415 cm which revealed that it is appropriate choice for shielding applications at lower energies. In summary, the prepared nanocomposites are more suitable for practical low-energy shielding applications rather than high-energy radiation. Also, the addition of Bi_2_O_3_ NPs improves resistance of unsaturated polyester for thermal degradation but, it decreases compressive strength of the composites.

## Data Availability

The data presented in this study are available on request from the corresponding author.
